# Synergistic effect of nano-Pt and Ni spine for HER in alkaline solution: hydrogen spillover from nano-Pt to Ni spine

**DOI:** 10.1038/s41598-018-21396-9

**Published:** 2018-02-14

**Authors:** Syed Asad Abbas, Seong-Hoon Kim, Muhammad Ibrahim Iqbal, Shoaib Muhammad, Won-Sub Yoon, Kwang-Deog Jung

**Affiliations:** 1 Center for Clean Energy and Chemical Engineering, Korea Institute of Science and Technology, Hwarangno 14-gil 5, Seongbuk-gu, Seoul 136-791 Republic of Korea; 2 Clean Energy and Chemical Engineering, University of Science and Technology, 217 Gajeong-ro Yuseong-gu, Daejeon, Republic of Korea; 3 Department of Energy Science, Sungkyunkwan University, Suwon, 440-746 South Korea

## Abstract

The design of active, stable, and cost-effective electrocatalysts for the H_2_ evolution reaction (HER) in alkaline conditions is important for electrochemical systems such as the chloro-alkaline process and H_2_ production. Here we report catalysts comprising Pt on Ni single crystalline spines (Pt/Ni-SP) with high activity and stability for HER in alkaline solution with proposed mechanism. The Pt/Ni-SP catalysts are prepared by dispersing platinum nanoparticles (1.7–3.1 nm) on the single-crystalline spines (Ni-SP) of Ni urchin-like particles. The size and coverage of Pt nanoparticles on Ni-SP are increased with increases in the Pt loading amount. X-ray diffraction, high-resolution transmission electron microscopy, X-ray photoelectron spectroscopy, and X-ray absorption spectroscopy are performed to observe the structure of the Pt/Ni-SP catalyst. The catalysts achieve the mass activity of 1.11 A mg^−1^_(Pt)_, comparing favorably to Pt/C catalysts with the mass activity of 0.33 A mg^−1^_(Pt)_ at 0.05 V overpotential. The Tafel slope of the Pt/Ni-SP catalyst is approximately 30 mV dec^−1^, similar to that of Pt, while Pt/Ni-SP is very stable in alkaline solution, like Ni. The synergistic effect of Pt/Ni-SP is ascribed to H spillover from Pt to Ni.

## Introduction

The H_2_ evolution reaction (HER) is very important step for the preparation of pure H_2_ from electrochemical water splitting using renewable energy^1^ as well as for energy storage purposes^2^. The biggest challenge is the development of catalyst materials for efficient H_2_ production with low costs and good electrochemical stability^3^. Carbon-supported Pt catalyst (Pt/C) is the reference material till date^4,5^, because it has high activitiy. However, its low stability and high cost have become practical limitation. For practical purposes, catalysts should be stable for several hundred hours^6^. The degradation mechanisms of Pt/C catalyst have been reported and summarized in the literature^7–9^ but the main reason of catalytic degradation in alkaline conditions for Pt/C as HER catalysts is the degradation of anchoring sites on the carbon support, which causes the Pt to detach from the support^10^. The interactions of degraded carbon supports and agglomerated detached Pt deteriorate the catalytic performance. Alternatively, certain non-carbon materials such as oxides^11^ and carbides^12^ of earth-abundant metals like Ti, W, and Mo exhibit good stability under high overpotential conditions, but show poor electrocatalytic activity because they have low conductivities^12–16^.

Stable multi-metallic nanoparticles are of great interest at present. Recently nanoframes of Pt_3_Ni^17^ and Pt–Ru–M (M = Ni, Fe, or Co) alloys^18^ have shown extraordinary results outperforming Pt alone, but Pt remains the basic component of these multi-metallic nanoparticles. In electrocatalysis, density functional theory (DFT) predicts better results for Pt skins^19,20^. The use of Pt skins substantially reduces the amount of Pt, but introduces new problems such as difficulties in manipulating the nanoscale elemental distribution^21^ and the surface segregation of Pt. Furthermore, Pt skin preparation requires high annealing temperatures that reduce the electrochemical active surface area by particle sintering^22^.

Rational catalytic design can boost H_2_ production through the careful selection of catalyst supports and the minimal usage of Pt. In this work, highly stable urchin-like Ni nanoparticles with single-crystalline spines^23^ (Ni-SPs) are used as support. Pt nanoparticles are uniformly dispersed over the Ni-SP with controllable coverage, size, and loading level. The Ni particles are highly conductive because of the high crystallinity of the Ni-SP. The preparation is simple, entailing Pt impregnation of Ni particles. The Pt nanoparticle-loaded Ni-SP catalyst exhibits much improved activity and stability in the HER in alkaline conditions compared to that of a commercial Pt/C catalyst (40% Pt on Vulcan XC72).

## Results and Discussion

### Deposition of Pt nanoparticles on urchin-like structures

**Figure 1:**
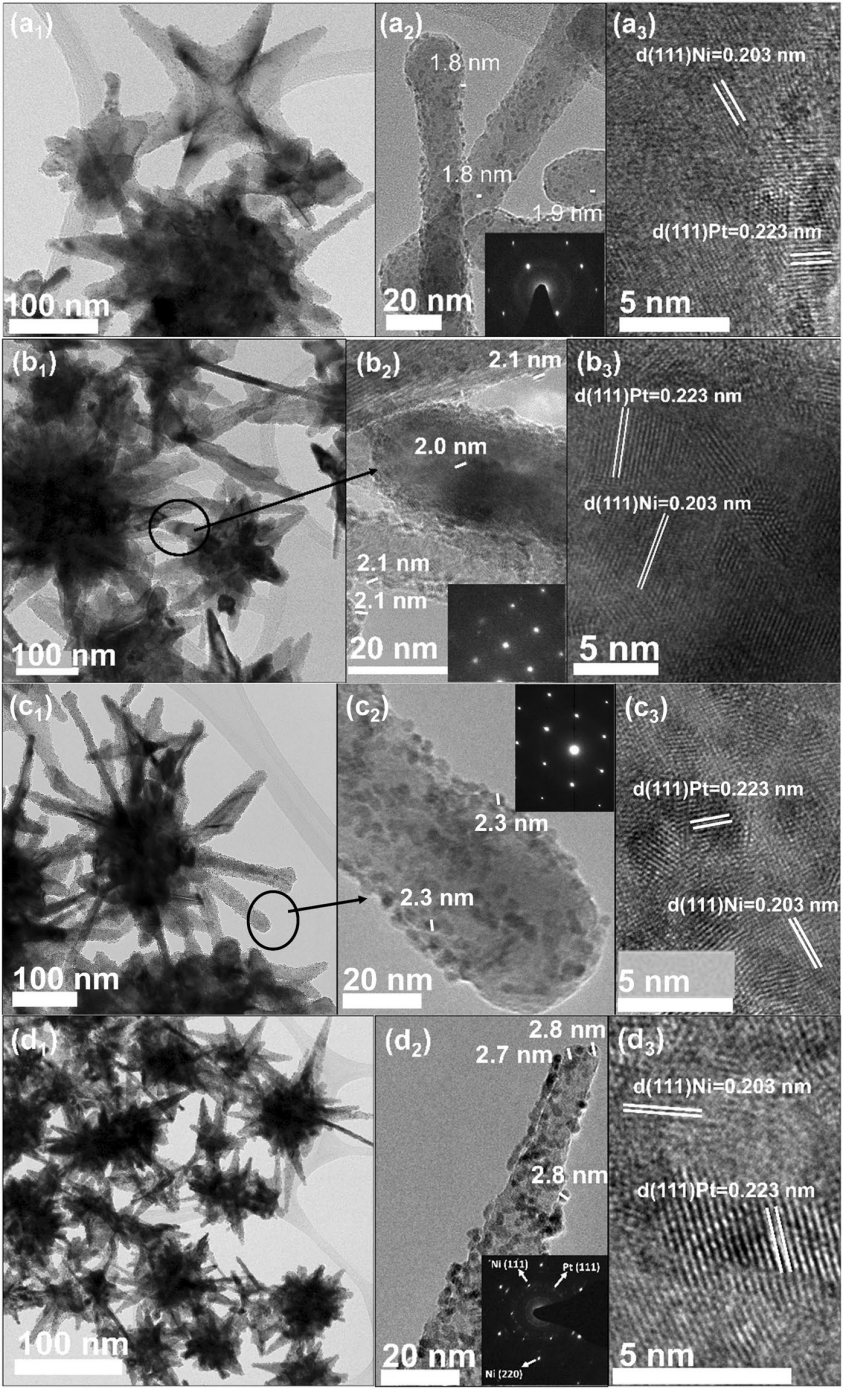
TEM images of the prepared catalysts: (**a**) 0.75Pt/Ni-SP, (**b**) 1Pt/Ni-SP, (**c**) 2Pt/Ni-SP, and (**d**) 5Pt/Ni-SP.

Pt islands of various sizes and thicknesses are assembled on urchin-like nickel structures. Four catalysts are prepared and named as 0.75Pt/Ni-SP, 1Pt/Ni-SP, 2Pt/Ni-SP, and 5Pt/Ni-SP for 0.75, 1, 2, and 5 mol% Pt loading on the surface of the Ni metal, respectively. The base Ni particles have urchin-like structures (Fig. 1(a1–d1)) and the Ni spines (SPs) of the particles show well-developed single-crystalline structures (Fig. 1(a3–d3)). The Pt particle size of the Pt/Ni-SP catalysts is increased as the Pt loading amount increases (Fig. 1(a2–d2)): ~1.8, ~2.0, ~2.3, and ~2.8 nm for 0.75Pt/Ni-SP, 1Pt/Ni-SP, 2Pt/Ni-SP, and 5Pt/Ni-SP, respectively. The transmission electron microscopy (TEM) images show the good dispersion of Pt particles on the Ni-SPs. A particle size histogram was constructed for all prepared catalysts (Fig. S2). The compositions and distributions of the Pt particles were also observed by high-angle annular dark-field scanning transmission electron microscopy (HAADF-STEM) images and corresponding energy-dispersive X-ray spectroscopy (EDS) mapping (Fig. S3), which showed Pt only on the surface of Ni. The EDS line profile in Fig. S4 also confirmed that the Pt particles were dispersed on the surface of Ni-SP. Table S1 shows the Pt loading amount as determined by an inductively coupled plasma optical emission spectroscopy (ICP-OES) analysis. The ICP-OES result confirmed that the designed Pt loading amount corresponded well to the actual loaded Pt amount.

**Figure 2:**
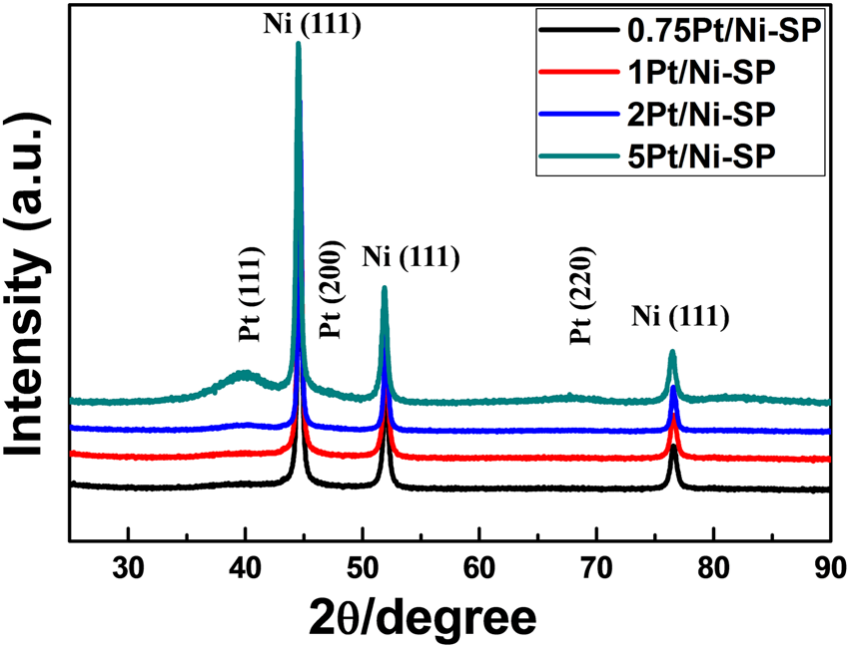
XRD patterns of prepared catalysts.

X-ray diffraction (XRD) analysis is performed to observe the crystallinities of the prepared catalysts, as shown in Fig. 2. From the XRD patterns, all the characteristic peaks of Ni (FCC) are very clear, showing good agreement with JCPDS 870712. The peaks at the 2θ values of 44.5°, 51.8°, and 76.4° are ascribed to the (111), (200), and (220) planes, respectively, indicating that no structural change occurs in the Ni particles during the process of Pt loading. The Pt (111) plane reflection is clear in the patterns of 5Pt/Ni-SP and 2Pt/Ni-SP, but not in those of 1Pt/Ni-SP and 0.75Pt/Ni-SP because of the low Pt loading amounts. Using Scherrer’s formula, the average particle sizes can be calculated. For the Ni urchin-like substrates, the average crystal size remains at 25.7 nm for all the prepared catalysts, while the crystal size of Pt increases as the Pt loading is increased. The Pt average crystal sizes are recorded as 1.67 and 2.8 nm for 2Pt/Ni-SP and 5Pt/Ni-SP, respectively, slightly smaller than the sizes observed in high-resolution TEM (HR-TEM) analysis.

X-ray photoelectron spectroscopy (XPS) measurements were performed to elucidate the surface compositions and chemistries of the prepared catalysts. The spectra of Ni 2*p* and Pt 4 *f* are shown in Fig. S1. The results are summarized in Table 1. For all the pristine samples, the binding energies for Pt 4*f*_7/2_ and Pt 4*f*_5/2_ are observed at 71.4 and 74.6 eV, respectively, corresponding to the Pt 4*f*_7/2_ and Pt 4*f*_5/2_ levels with a binding energy difference of 3.2 eV and indicating the metallic state of Pt^18^. After the deconvolution of Ni 2*p*_3/2_, three peaks are observed at 852.6 eV, 854.2 eV, and 856.2 eV, corresponding to Ni^(0)^, NiO, and Ni(OH)_2_, respectively^24,25^. The content of Ni(OH)_2_ is near 50% on the surfaces of all prepared samples; that of NiO also remains consistent at approximately 30%. However, the metallic content slightly decreases as the amount of Pt loading is increased, confirming that the Pt is attached to Ni metallic sites. The XPS analysis of the used catalyst after reaction of 50 ks at −1.5 V vs. Hg/HgO showed no NiO peak (Fig. S11), because the passivated Ni(OH)_2_ and NiO on the surface of Ni were reduced to metallic Ni during HER in alkaline conditions, as described by MacDougall *et al*.^26^. The XPS analysis of the used catalyst also confirmed the reduction of Pt oxides to Pt metal; after deconvolution, no oxide peaks were observed for the loaded Pt catalyst. On the surface of the catalyst, the amount of Pt atoms increases as the loading of Pt is increased; Pt atoms occupy 15%, 30%, and 47% of the Ni surface for 1Pt/Ni-SP, 2Pt/Ni-SP, and 5Pt/Ni-SP, respectively.

**Table 1:** XPS analysis results for Pt 4*f* and Ni 3*p*.

Sample	Pt^(0)^ 4*f*_7/2_	Pt^(+2)^4*f*_7/2_	Metallic %	Ni^(0)^ 2*p*_3/2_	Ni^(+2)^ 2*p*_3/2_ (NiO)	Ni^(0)^ 2*p*_3/2_ Ni(OH)_2_	Chi squared values	Metallic %
0.75Pt/Ni-SP	71.4	73.8	76	852.6	854.2	856.2	0.82	22
1Pt/Ni-SP	71.4	73.8	75	852.6	854.2	856.2	0.79	20
2Pt/Ni-SP	71.4	73.8	75	852.6	854.2	856.2	0.38	16
5Pt/Ni-SP	71.4	73.8	75	852.6	854.2	856.2	0.57	15
1Pt/Ni-SP Used Catalyst	71.1	—	100	853.1	—	856.2	0.36	10

**Figure 3:**
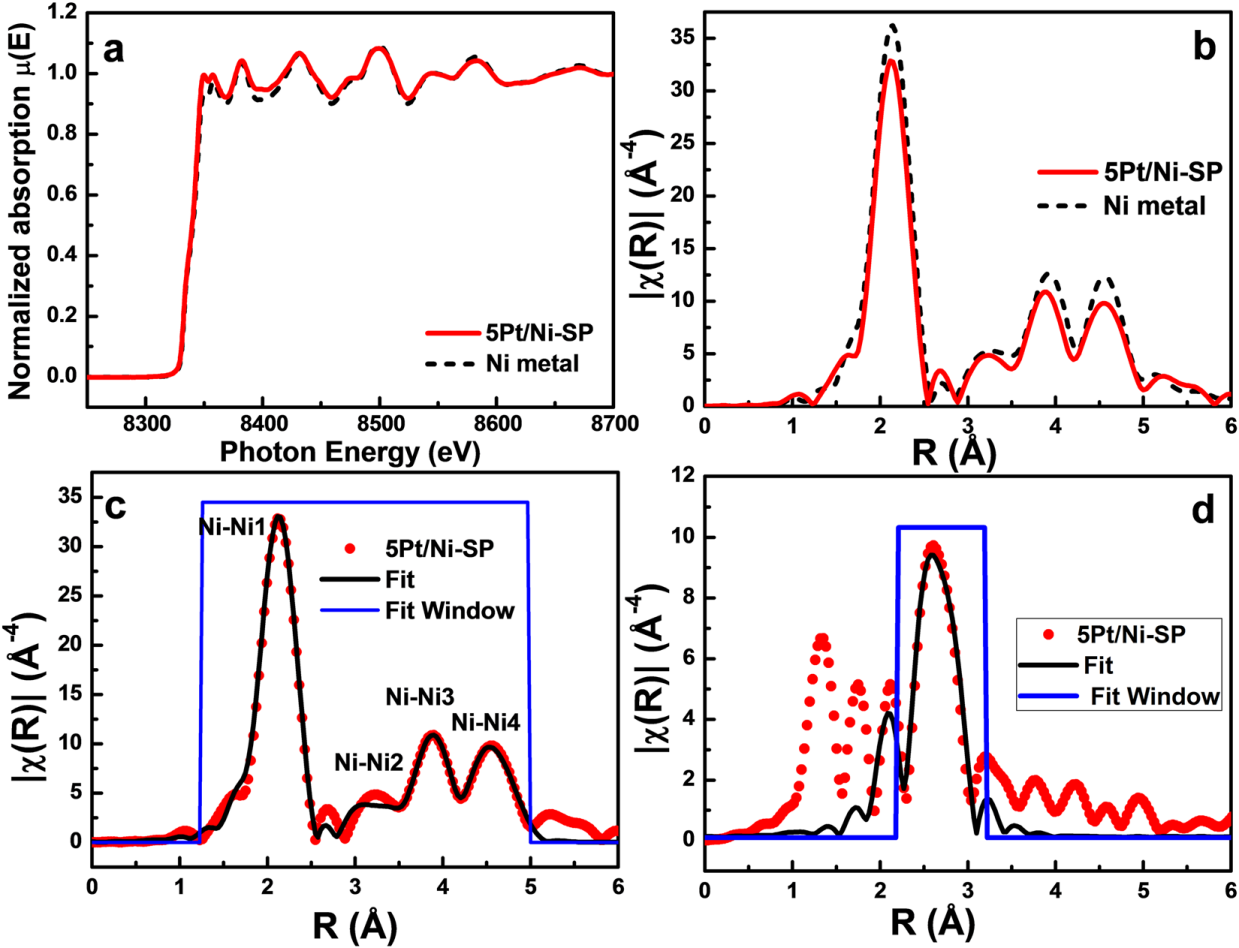
XAS characterizations of 5Pt/Ni-SP with Ni K-edge and Pt with L_3_-edge. (**a**) Comparison of XANES spectrum with 5Pt/Ni-SP and Ni foil, (**b**) comparison of EXAFS spectrum with 5Pt/Ni-SP and Ni foil, (**c**) Ni K-edge fitted EXAFS spectrum of 5Pt/Ni-SP and (**d**) Pt L_3_-edge fitted EXAFS spectrum of first coordination shell for 5Pt/Ni-SP.

X-ray absorption near-edge structure (XANES) and extended X-ray absorption fine-structure (EXAFS) measurements are performed to obtain insight on the local structure of the Pt-loaded Ni-SP catalyst in order to determine the detailed mechanism of electrochemical HER on the catalyst. XANES provides information on the oxidation states based on edge positions and white line intensity. Figures 3(a) and S5(a) clearly exhibit no substantial change in the edge positions of the prepared Ni catalysts compared to that of standard Ni metal. The Ni K-edge position remains at 8333–8334 (eV), indicating a small amount of Ni^+ ^^2^ present on the surface of Ni, as observed in the XPS analysis. For large amounts of NiO on the surface of Ni, the absorption K-edge would be shifted to a higher value reaching 8344–8347 (eV)^27^. The white line intensities, positions of the Ni K-edges, and shapes of the EXAFS spectra for all Pt/Ni-SP samples exhibit no noticeable changes, as shown in Fig. S5 (a,b), indicating that the electronic and atomic structures are similar for all samples.

X-ray absorption spectroscopy (XAS) studies were mainly performed to observe the local structures and oxidation states of the Ni and Pt atoms in the proposed catalyst. In Fig. 3(a), and Fig. 3(b), Ni K-edge XANES and corresponding EXAFS spectra of the 5Pt/Ni-SP catalyst are compared with those of Ni metal, respectively. No noticeable differences appear between these two sets of spectra. Therefore, the EXAFS spectra of the Ni and Pt atoms in the 5Pt/Ni-SP catalyst are fitted with theoretical standards of the Ni K-edge, as shown in Fig. 3(c), and the Pt L_3_-edge, as shown in Fig. 3(d). In the Ni K-edge EXAFS spectra, the first prominent peak appears at 2.11 Å. The fitting results of the Ni K-edge EXAFS show that this peak represents the Ni–Ni coordination shell with the bond length of 2.483 Å^28^. The other low-intensity peaks, labeled as Ni–Ni2, Ni–Ni3, and Ni–Ni4, are present in the Ni K-edge EXAFS spectra at higher R values and represent higher Ni–Ni coordination shells in the Ni metal. The Ni K-edge EXAFS spectrum shows good agreement with that of Ni metal and no representative peak for Ni–Pt bonds is observed. The Pt L_3_-edge fit with Pt standard metal also shows good agreement. The Pt L_3_-edge EXAFS fitting for 5Pt/Ni-SP is shown in Fig. 3d, with results presented in Table 2. The coordination number of Pt–Pt in the first shell is 8.34, less than the theoretical value of 12. This low coordination number for Pt is expected because of the very small size of Pt particles^29^ and corroborates the HR-TEM analysis as shown in Fig. 1. The Pt–Pt bond length in 5Pt/Ni-SP is 2.7491 Å, which is very close to the Pt–Pt bond length in Pt metal and significantly longer than the Pt–Ni bond length of 2.66 Å^30^. From the above discussion, we can conclude that the Pt particles at the surface of Ni are mainly in the separate metallic state, without alloy formation.

**Table 2:** Results obtained from the EXAFS fits of the 5Pt/Ni-SP for Ni K-edge and 5Pt/Ni-SP for Pt L_3_-edge.

Sample	Path	Coordination No.	σ^2^	Radial distance (Å)
5Pt/Ni-SP	Ni-Ni1	12	0.00626	2.4830
	Ni-Ni2	6	0.00936	3.4882
	Ni-Ni3	24	0.00971	4.3156
	Ni-Ni4	24	0.01017	5.0467
5Pt/Ni-SP	Pt-Pt1	8.34	0.00670	2.7491

Value of S_0_^2^ was used as 0.7 and 0.8 for Ni and Pt K-edge EXAFS spectrum fitting. Reduced chi-square values that represent the goodness-of-fit are 65.27 and 5.27 for Ni and Pt K-edge EXAFS spectrum fitting.

### HER Performance

**Figure 4:**
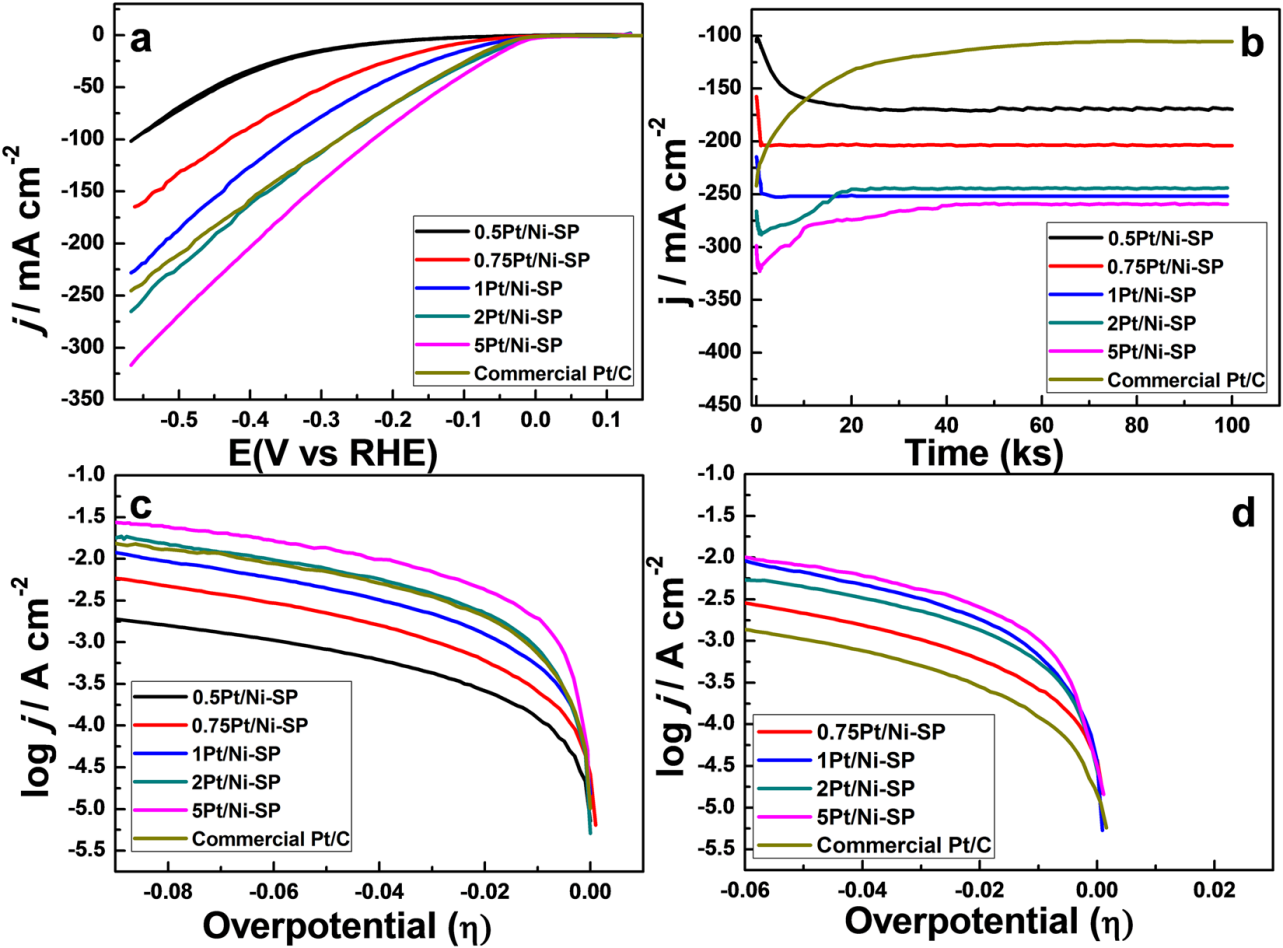
HER activity (**a**) LSV at 50 mV s^−1^ in 1-M NaOH, (**b**) Stability test for 100 ks at −1.5 V vs. Hg/HgO in 1-M NaOH, (**c**) Initial Tafel slopes at 1 mV s^−1^ in 1-M NaOH, (**d**) Tafel slopes after 50 ks of stability testing at −1.5 V vs. Hg/HgO of the prepared catalysts in 1-M NaOH.

The HER activities of the prepared catalysts were investigated using linear-sweep voltammetry (LSV) at the scan rate of 50 mV·s^−1^ from 0.15 V vs. a reversible hydrogen electrode (RHE) to −0.57 V vs. RHE at room temperature in 1 M NaOH solution. The same total weights of catalysts were loaded on glassy carbon, with the net weight of Pt varying depending on the amount of Pt loading on the Ni substrate. The Pt loading amount on the commercial Pt/C catalyst (40% Pt on Vulcan XC 72) was equal to the Pt loading on the 5Pt/Ni-SP catalyst. The specific activity of the 5Pt/Ni-SP catalyst was much higher than that of the Pt/C catalyst; meanwhile, that of the 2Pt/Ni-SP was comparable to that of the Pt/C catalyst. The mass activity of 2Pt/Ni-SP was approximately 3.15 times higher than that of the Pt/C catalyst, as 2Pt/Ni-SP contains 2.5 times less Pt than the Pt/C catalyst. The specific activity of the prepared Pt/Ni-SP catalysts is increased as the Pt loading amount is increased, as shown in Fig. 4(a). The initial activities of 5Pt/Ni-SP, 2Pt/Ni-SP, 1Pt/Ni-SP, and 0.75Pt/Ni-SP are −317 mA cm^−2^, −265 mA cm^−2^, −228 mA cm^−2^, and −164 mA cm^−2^, respectively, while that of Pt/C is −245 mA cm^−2^.

The stability of a catalyst is among the most important features from a practical perspective. The stabilities of the prepared samples are investigated at the potential of −1.5 V vs Hg/HgO for 100 ks (Fig. 4(b)). Commercial Pt/C begins to deactivate; at 10 ks, 34% of the initial activity is lost, as reported in the literature^10^, while 57% of the initial activity is lost overall before 100 ks of the reaction. Huajie *et al*. observed a 39.4% loss in activity for the first 4 ks of a 20% commercial Pt/C catalyst^31^.

For the Pt/Ni-SP catalysts, all specimens show initial increases in activity, a common phenomenon for the Ni-based HER catalysts because surface NiO present on the surface is reduced, as has been explained previously^26^. The reduction of surface NiO to Ni metal provides more active surface area for electrocatalysis and more vacant sites for H_2_ diffusion. XPS analysis of the used catalyst also confirmed the absence of NiO on the surface of the catalysts (Fig. S11). The Pt/Ni-SP catalysts with Pt loading >1 mol% show some deactivation after a few kiloseconds. To investigate this deactivation, TEM analysis of the used catalyst was performed; agglomerations of Pt particles on the Ni base are found for 2Pt/Ni-SP (Fig. S9) and 5Pt/Ni-SP (Fig. S10), while in the low-loading catalysts of 1Pt/Ni-SP (Fig. S8) and 0.75Pt/Ni-SP (Fig. S7), no agglomeration was observed. In 5Pt/Ni-SP and 2Pt/Ni-SP, after this initial degradation, no further deactivation occurs before 100 ks. Therefore, the initial deactivation of the 2Pt/Ni-SP and 5Pt/Ni-SP catalysts is ascribed to Pt sintering.

Tafel slopes and exchange current densities of all the Pt/Ni-SP catalysts are calculated from Fig. 4(c) and tabulated in Table 3. From the values, increased loading of Pt is clearly correlated with increased exchange current values. The Tafel slope is a potential-dependent property^32^, so all the Tafel slopes were calculated in the range of 10 to 30 mV of applied overpotential. The Tafel slope value of the Pt/Ni-SP catalysts with the Pt amounts of (0.75, 1, 2, and 5) mol% is ~30 mV dec^−1^, while that of the 0.5Pt/Ni-SP with 0.5 mol% Pt loading is ~115 mV dec^−1^. The Tafel slopes of Ni-SP and Ni catalysts have been reported as ~120 mV dec^−1^ previously^23,33^; therefore, 0.5Pt/Ni-SP behaves like a metallic Ni catalyst. The Tafel slope of ~30 mV dec^−1^ with a Ni catalyst is impossible at the low overpotential of 30 mV, as described by Lasia^34^. The chemisorption energy of Pt that results in minimum Gibbs’s free energy, H_2_ adsorption on Pt, is essentially thermoneutral and the dissociation of water is much easier on Pt surfaces than on Ni^35^. The adsorption of H_2_ on Ni is endothermic reaction^36^ and more energy is required to overcome the H_2_ adsorption barrier than that on Pt^37^; therefore, only Pt can show the low Tafel slope of ~30 mV dec^−1^ in the low overpotential range of 10 to 30 mV. Hence, it is plausible that the Pt/Ni-SP catalysts with Pt amounts >0.5 mol% behave like Pt in mechanistic aspects. From the Tafel slopes, the Tafel recombination reaction is the rate-determining step for HER in both the Pt/C and Pt/Ni-SP catalysts^38^ with Pt amounts >0.5 mol%, while a Volmer reaction is the rate-determining step for Ni and 0.5Pt/Ni-SP. To confirm the stable activity of the catalysts, the kinetic parameters (Tafel slope and exchange current densities) are also observed after 50 ks of HER at −1.5 V vs. Hg/HgO (Fig. 4(d)) and tabulated in Table 3; these kinetic parameters corroborate the stability test results. The exchange current densities of the low-Pt-loaded catalysts (0.75Pt/Ni-SP and 1Pt/Ni-SP) are increased because the catalyst is activated by the reduction of NiO and Ni(OH)_2_ from the surface of Ni-SP; meanwhile, the exchange current densities of 2Pt/Ni-SP and 5Pt/Ni-SP are decreased after 50 ks of reaction by Pt agglomeration-induced deactivation.

**Table 3:** Catalytic performance of prepared catalysts in 1-M NaOH solution for HER.

Sample	Initial Tafel slope mV/dec	Tafel slope after 50 ks mV/dec	Initial exchange current mA cm^−2^	Exchange current after 50 ks mA cm^−2^	Electrochemical surface area m^2^ g^−1^_(Pt)_	Overpotential (η) mV for 10 mA cm^−2^
0.5Pt/Ni-SP	115		0.199			250
0.75Pt/Ni-SP	32	32	0.21	0.25	89	125
1Pt/Ni-SP	28.3	31	0.40	0.53	97	76
2Pt/Ni-SP	28.5	32.5	0.75	0.41	72	42
5Pt/Ni-SP	29.1	33	1.25	0.72	63	32
Commercial Pt/C	30	30	0.62	0.12	34	53

**Figure 5:**
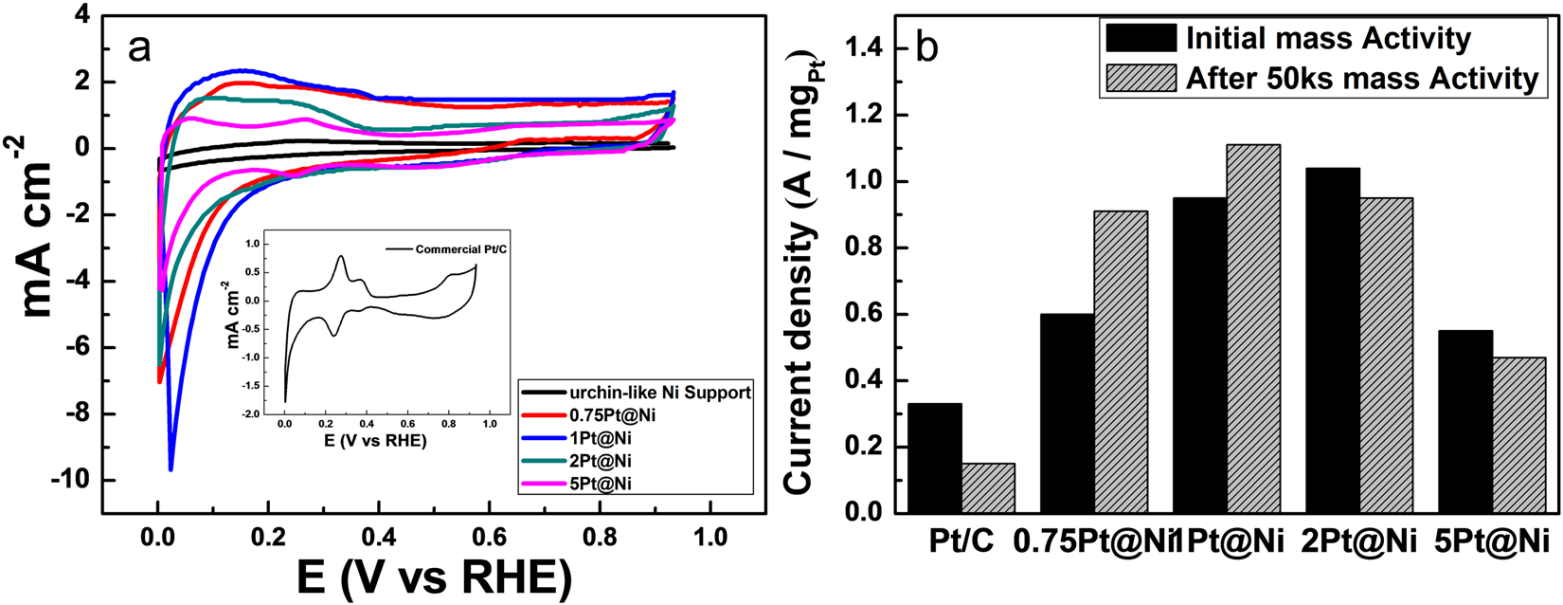
(**a**) CV analysis in the range of the H_2_ adsorption–desorption region at 50 mV s^−1^ in 1-M NaOH, (**b**) mass activity of prepared catalysts at the overpotential of 0.05 V.

Cyclic voltammetry (CV) analysis is performed to observe the H_2_ adsorption and desorption phenomena (Fig. 5(a)). For CV analysis, the current is normalized to the total weight of Pt loaded, allowing clear observation. In the range of H_2_ adsorption and desorption, urchin-like Ni supports show no current peaks and the double-layer current is also very low. All the Pt/Ni-SP catalysts exhibit increases in electrochemical surface area (ECSA) as the amount of Pt loading approaches 1 Pt mol%; the ECSA decreases with further increases in Pt loading. The ECSA of 5Pt/Ni-SP with the average Pt particle size of 2.8 nm is much larger than that of Pt/C with the average Pt particle size of 2.8 nm and equal Pt loading. The 5Pt/Ni-SP catalyst shows two different peak positions for the under- and overpotential desorption steps, but for Pt/Ni-SP catalysts with lower Pt loadings, both peaks are merged and broadened, indicating much larger ECSA while the Pt particle size is decreased as well. However, distinctive H_2_ desorption peaks on Pt/C with the Pt particle size of 2.2 nm have been reported in the literature^39^. Therefore, the high ESCA and disappearance of these distinctive H_2_ desorption peaks cannot be attributed to the small Pt particles alone. It is plausible that H spillover may continuously generate vacancy active sites at the Pt surface; the total ECSA would increase with increasing H spillover^40^. The H spillover mechanism is supported by the diffusional desorption peak of H prior to the reversible desorption peaks of H_2_^41^. H desorption begins at much lower voltages on the Pt/Ni-SP catalysts than on the Pt/C catalyst, at 0.27 and 0.37 V vs. RHE, respectively, as shown in Fig. 5(a). Mavrikakis *et al*. have explained the relation between the binding energy of adsorbed H and the diffusion activation energy; the diffusional barrier is approximately12% of the binding energy of the adsorbed state^42^. At 298 K, the activation energy of surface H diffusion on Pt ranges from 10 kJ mol^−1^ to 25 kJ mol^−1^, yielding the diffusion coefficient of 10^−5^ cm^2^ s^−1^ and causing instantaneous H migration at room temperature; by these steps, H spillover can be rationalized^43^. Furthermore, if the rate-determining step is the Tafel recombination reaction, H spillover can boost H_2_ production^44^. Electrochemical impedance spectroscopy is also performed and discussed in supplementary information (Fig. S13 and Table S2) that corroborates with the kinetic parameters obtained from Tafel slope.

The Mass activities of the prepared catalysts at the overpotential of 0.05 V vs. RHE are also observed (Fig. 5(b)) and show good agreement with the CV results for the ECSA. Overpotential of 0.05 V is selected for the mass activity measurement because the onset potential of Ni-SP was observed at 0.078 mV, so the activity contribution of Ni-SP at 0.05 V is negligible. The Pt-based initial mass activities of the prepared catalysts are 0.33 A mg^−1^, 0.6 A mg^−1^, 0.95 A mg^−1^, 1.04 A mg^−1^, and 0.55 A mg^−1^ for Pt/C, 0.75Pt/Ni-SP, 1Pt/Ni-SP, 2Pt/Ni-SP, and 5Pt/Ni-SP, respectively. After the 50 ks of stability testing, these values have changed to 0.15 A mg^−1^, 0.91 A mg^−1^, 1.11 A mg^−1^, 0.95 A mg^−1^, and 0.47 A mg^−1^, showing the same trend as the ECSA. In addition, the Ni-SP support enhances the stability of the loaded Pt. From the above data we can conclude that, for the best stability and activity with the least amount of Pt, 1Pt/Ni-SP is the best catalyst among those designed and tested here.

## Conclusion

Here we have designed a catalyst by the addition of Pt on crystalline Ni spines in ethylene glycol. The sizes of the Pt particles on the Ni-SP supports ranged from 1.8 nm to 2.8 nm, depending on the amount of Pt loading. The mass activity was optimized with the 1 mol% Pt, 1Pt/Ni-SP catalyst have much higher mass activity than that of the commercial Pt/C catalyst. TEM and EXAFS analysis clearly showed that the isolated Pt particles were well dispersed on the crystalline Ni-SP supports. The rate-determining step for HER over the Pt/Ni-SP catalysts was changed from the Volmer reaction to the Tafel recombination reaction as the Pt loading on the Ni-SP was increased. Furthermore, a large ECSA was observed with the 1Pt/Ni-SP catalyst, attributed to H spillover from Pt to Ni. Ni metal has higher stability but lower activity than Pt. The lower activity of Ni arises from the slow rate of water dissociation (the Volmer step); the split H atoms on Pt and Ni recombine on Ni to produce H_2_. Therefore, it can be concluded that the small Pt particles on Ni-SP enhanced the catalyst mass activity, which was further enhanced by H spillover from the Pt surface to the crystalline Ni surface. No Pt agglomeration was observed with the 1Pt/Ni-SP catalyst, even after 50 ks of HER. By combining theory with practical results, the mass activity of Pt was increased from 0.33 A mg^−1^ in the commercial Pt catalyst to 1.11 A mg^−1^ for 1Pt/Ni-SP.

## Methods

### Chemicals

Potassium hexachloroplatinate (98% K_2_PtCl_6_), Ni II acetyleneacetonate (Ni(acac)_2_ 95%), oleylamine (OAm 70%), ethanol (99.5%), and ethylene glycol (99%) were purchased from Sigma–Aldrich Co., Ltd. The Pt/C catalyst was purchased from Premetek (40% Pt on Vulcan XC 72).

### Pt nanoparticle deposition on Urchin-like Ni structures

For the synthesis, 58.7 mg (1 mmol) of urchin-like Ni nanoparticles and 0.0075, 0.01, 0.02, and 0.05 mmol of potassium hexachloroplatinate were dissolved in 20 mL of ethylene glycol and held for 3 h in a rotary reactor at room temperature to allow the complete dissolution and impregnation of Pt precursor on the Ni nanoparticles. After 5 min of bath sonication, the homogeneous solution was heated in a water bath at 90 °C for 1 h. After the reaction, the ethylene glycol was removed by centrifugation at 8000 rpm and washed with deionized water several times to remove residual ethylene glycol; the product was then dried in a vacuum oven at 25 °C for 5 h. For the deposition of Pt nanoparticles on the urchin-like Ni nanoparticles, the NPs were prepared in one batch. For ease of description, the prepared Pt-loaded catalysts are named as 0.75Pt/Ni-SP, 1Pt/Ni-SP, 2Pt/Ni-SP, and 5Pt/Ni-SP for 0.75, 1, 2, and 5 mol% Pt loading, respectively.

### Characterization

Transmission electron microscopy (TEM) and high-resolution transmission electron microscopy (HR-TEM) were performed using a FEI Tecnai TEM G^2^ (accelerating voltage of 200 kV). Powder X-ray diffraction (XRD) was performed using a Bruker D8 Avance diffractometer using Cu Kα radiation (λ = 1.5406 Å). XPS analyses were recorded on a PHI 5000 VersaProbe (Ulvac-PHI) with the background pressure of 2 × 10^−7^ Pa and the spot size of 100 μm × 100 μm at an angle of 45°, wide scan pass energy of 117.4 eV and narrow scan pass energy of 46.95 eV. The concentrations of the catalysts were analyzed using Thermoscientific iCAP7000. X-ray absorption (XAS) data was collected at the 1D KIST-PAL beamline in the Pohang light source (PLS-II). The storage ring was operated at 2.5 GeV with an injection current of 360 mA. The intensity of the incident X-ray absorption *I*_o_ was monitored with a N_2_-filled ionization chamber. The spectral energies were calibrated by using the first inflection points of Ni (8333 eV) and Pt (11564 eV) metal foil spectra as references. XANES data preprocessing and EXAFS data fitting with theory were performed by using the IFEFFIT-based program Demeter based on FEFF code^45,46^.

### Electrochemical measurements

All the electrochemical measurements were performed in a three-electrode cell at room temperature (25 °C), with Hg/HgO as the reference electrode and a Pt foil as the counter electrode, respectively. The working electrode was prepared with a glassy carbon rotating disk of 5 mm in diameter as the substrate. Typically, a mixture containing 4.0 mg of the electrocatalyst in a solvent of 0.9 mL Water and 0.1 mL 5 wt% Nafion solution (5 wt%, DuPont, USA) was ultrasonicated for 5 minutes to obtain a well-dispersed ink. The catalyst ink was then quantitatively transferred onto the surface of the glassy carbon electrode and dried at room temperature to obtain a thin film of the catalyst. The total catalyst loading was 0.204 mg cm^−2^. The Pt/C commercial catalyst was deposited at 0.031 mg cm^−2^, equivalent to the amount of Pt in the 5Pt/Ni-SP catalyst. The electrochemical tests were performed in Ar-saturated 1 M NaOH aqueous solution at room temperature. CV scanning was performed at the potential scan rate of 50 mV s^−1^ in the range of 0.005 V to 0.93 V vs. RHE to determine the electrochemical surface areas. Linear sweeps to obtain the Tafel slopes were performed at the potential scan rate of 1 mV s^−1^ in the range of −60 mV to 0 mV vs RHE while the hydrogen evolution reaction polarization curve was obtained at the scan rate of 50 mVs^−1^ in the range of 0.2 V to −0.57 V vs Hg/HgO. Chronoamperometric analysis was also performed at −1.5 V (vs. Hg/HgO) for 50 ks and 100 ks. All the electrochemical studies were performed on an (Ivium Technologies, Iviumstat), coupled with a rotating-disk electrode (PINE-AFMARCE-5 mm dia).

## Electronic supplementary material

Supplementary Information
