# Supplementary material for: Synergistic effect of nano-Pt and Ni spine for HER in alkaline solution: hydrogen spillover from nano-Pt to Ni spine
Source: Sci Rep. 2018 Feb 14;8:2986. doi: 10.1038/s41598-018-21396-9 (PMC5813055; doi:10.1038/s41598-018-21396-9)
Supplement: Supplementary file 1 — Supplementary Information [file 41598_2018_21396_MOESM1_ESM.pdf]

Supplementary Information

Synergistic effect of nano-Pt and Ni spine for HER in alkaline solution: hydrogen spillover from nano-Pt to Ni spine

Syed Asad Abbas[a,b], Seong-Hoon Kim[a], Muhammad Ibrahim Iqbal[a,b], Shoaib Muhammad[c], Won-Sub Yoon[c], Kwang-Deog Jung[a,b*]

[a] *Center for Clean Energy and Chemical Engineering, Korea Institute of Science and Technology, Hwarangno 14-gil 5, Seongbuk-gu, Seoul 136-791, Republic of Korea*

[b] *Clean Energy and Chemical Engineering, University of Science and Technology, 217 Gajeong-ro Yuseong-gu, Daejeon, Republic of Korea*

[c] *Department of Energy Science, Sungkyunkwan University, Suwon 440-746, South Korea*

*Author to whom all correspondence should be addressed

Tel: +822-958-5218, Fax: +822-958-5219

E-mail: jkdcat@kist.re.kr

Figure S1

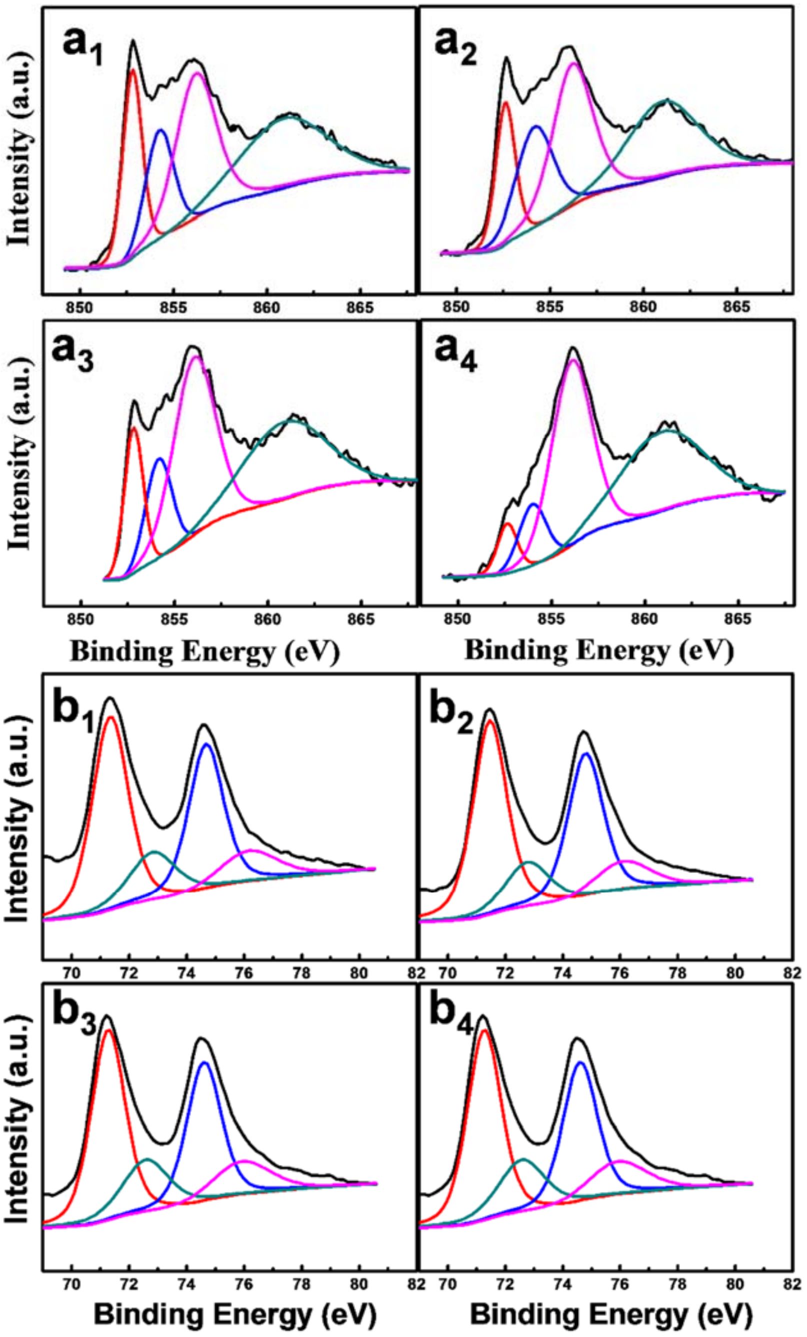

Fig. S1. XPS analysis of prepared catalysts: Ni for (a_1) 0.75Pt/Ni-SP, (a_2) 1Pt/Ni-SP, (a_3) 2Pt/Ni, and (a_4) 5Pt/Ni-SP; Pt for (b_1) 0.75Pt/Ni-SP, (b_2) 1Pt/Ni-SP, (b_3) 2Pt/Ni-SP, and (b_4) 5Pt/Ni-SP.

Figure S2.

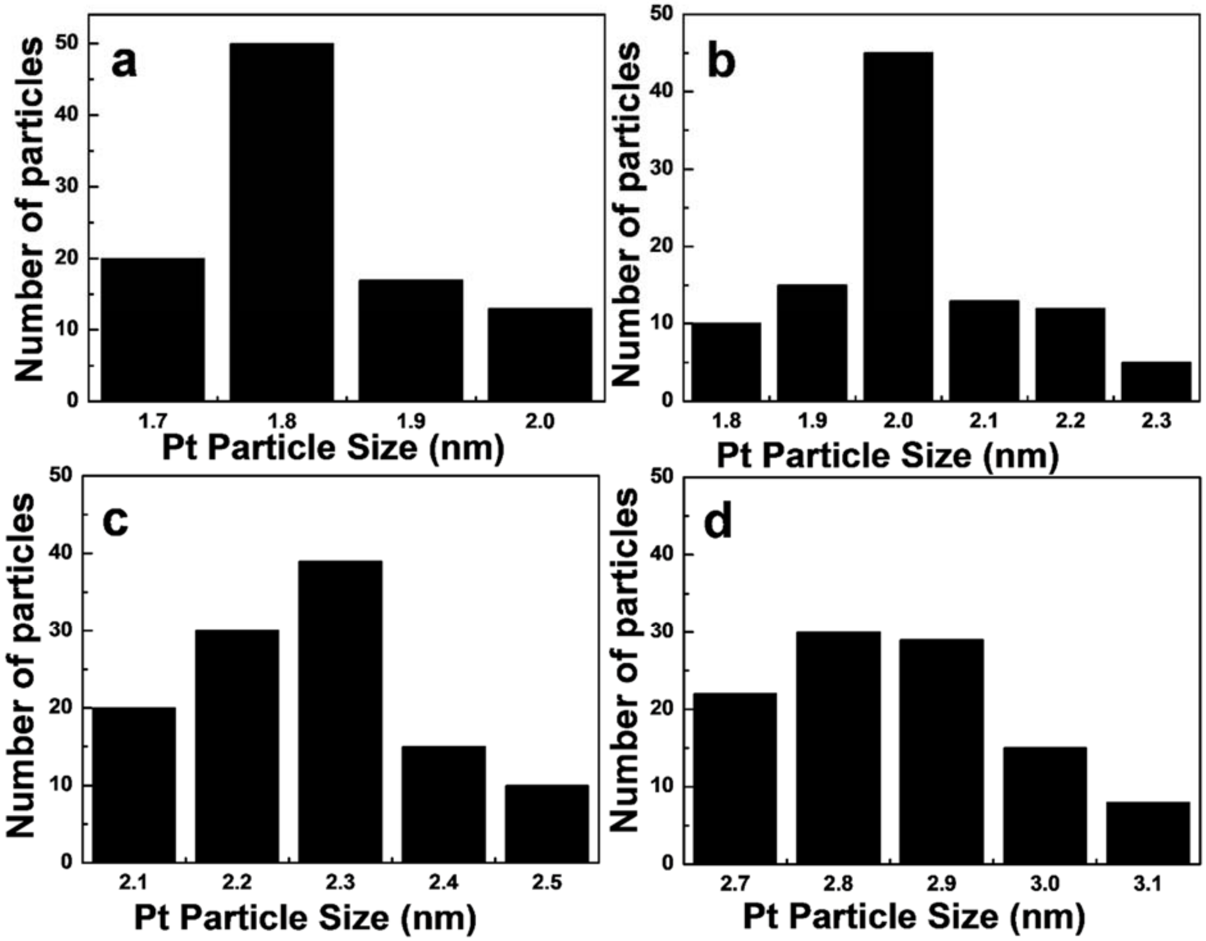

Fig. S2. Particle size distribution of Pt particles on the Ni base for (a) 0.75Pt/Ni-SP, (b) 1Pt/Ni-SP, (c) 2Pt/Ni-SP, and (d) 5Pt/Ni-SP.

Figure S3.

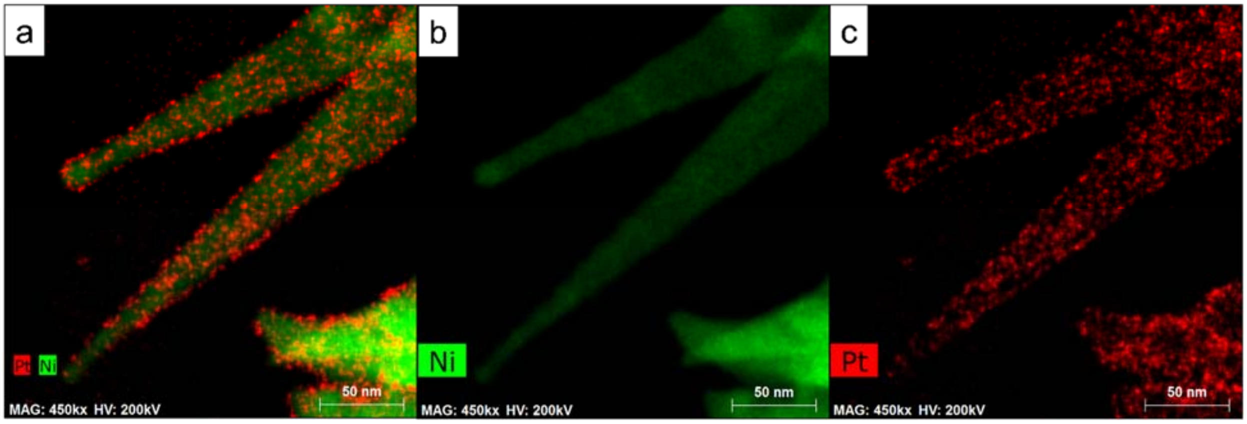

Fig. S3. HAADF images of 1Pt/Ni-SP.

Figure S4

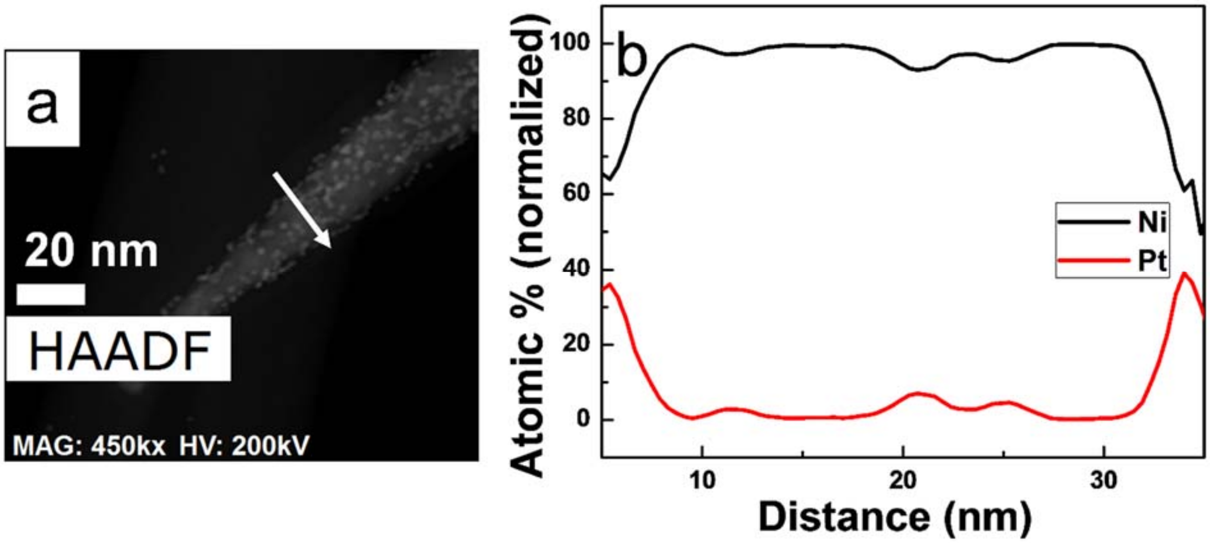

Fig. S4. EDS line profile crossing the Pt-loaded Ni structure

Figure S5

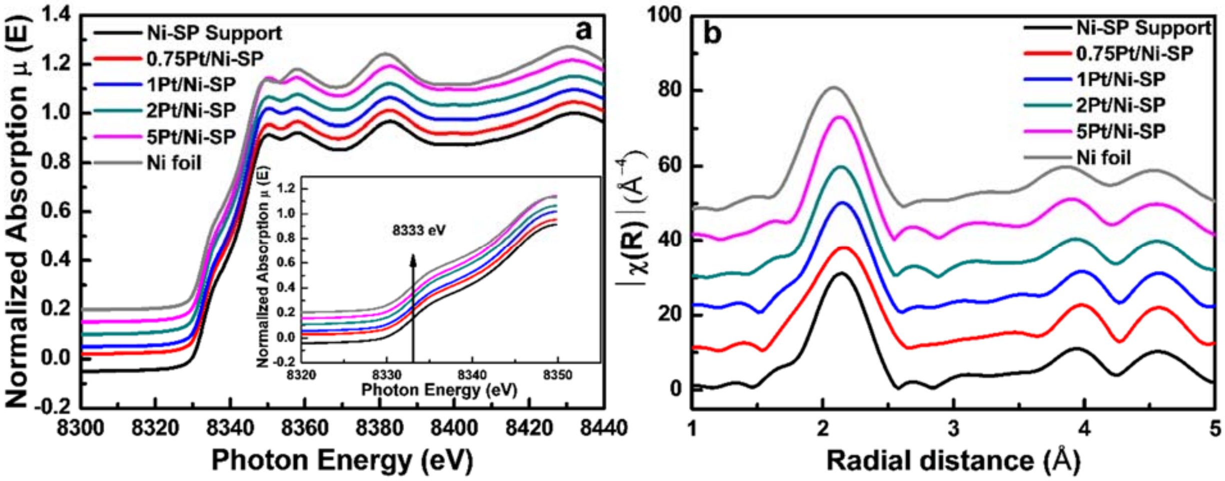

Fig. S5. XAS of prepared catalysts: (a) Ni K-edge XANES spectrum and (b) Fourier-transform EXAFS spectrum of Ni K-edge.

Figure S6

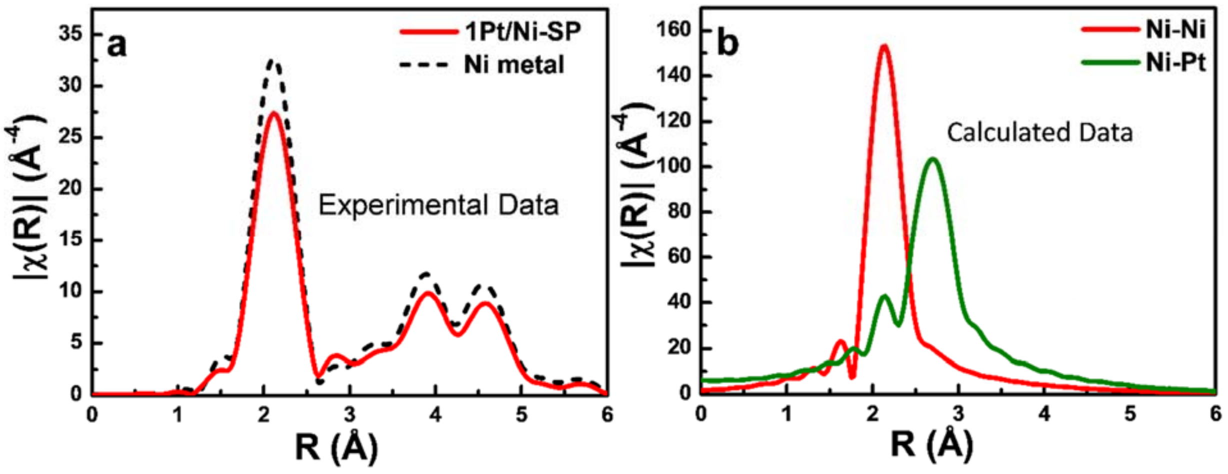

Fig. S6. Theoretical EXAFS data for the first coordination shell observation: (a) next neighbor if Ni atom is bonded to Ni atom; and (b) next neighbor for Ni–Pt bond.

Figure S7

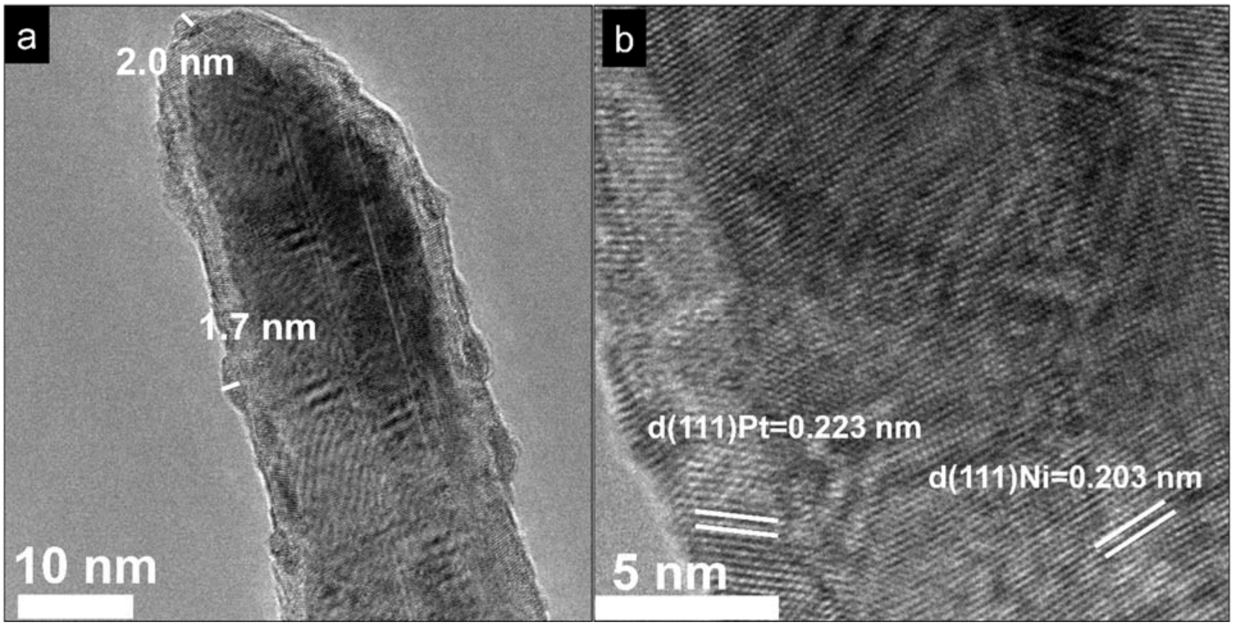

Fig. S7. TEM and HR-TEM of 0.75Pt/Ni-SP, used for HER analysis for 50 ks at −1.5 V vs. Hg/HgO in 1-M NaOH.

Figure S8

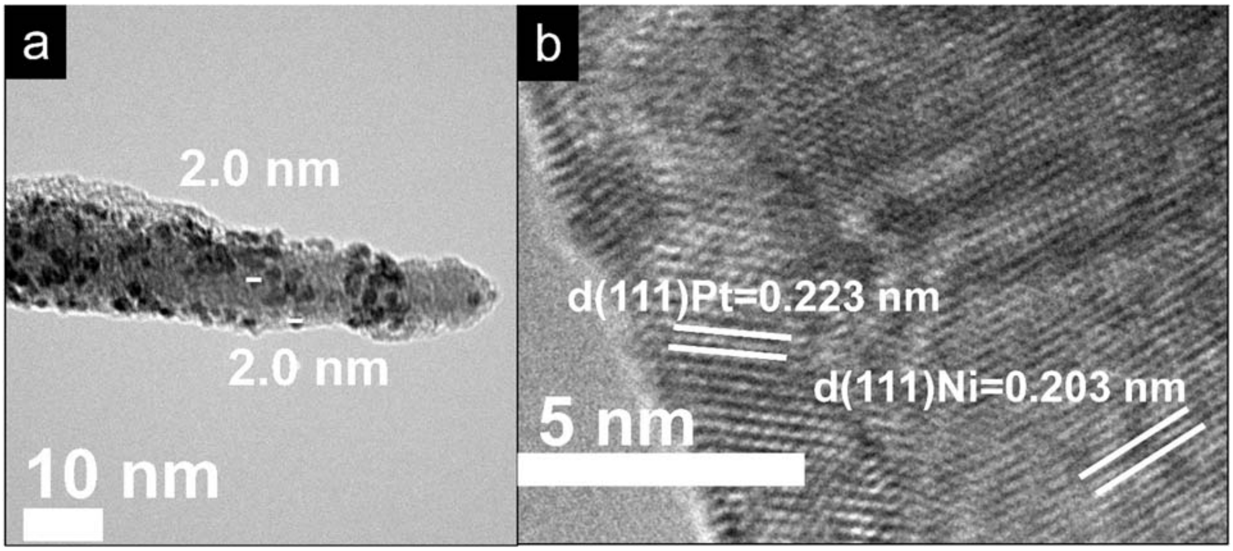

Fig. S8. TEM and HR-TEM of 1Pt/Ni-SP, used for HER analysis for 50 ks at −1.5 V vs. Hg/HgO in 1-M NaOH.

Figure S9.

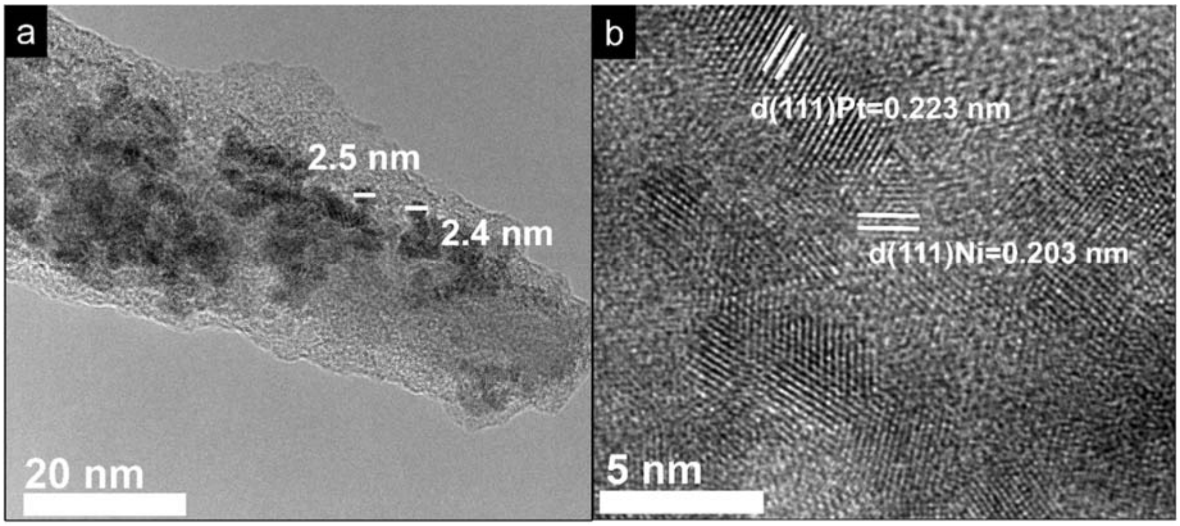

Fig. S9. TEM and HR-TEM of 2Pt/Ni-SP, used for HER analysis for 50 ks at −1.5 V vs. Hg/HgO in 1-M NaOH.

Figure S10.

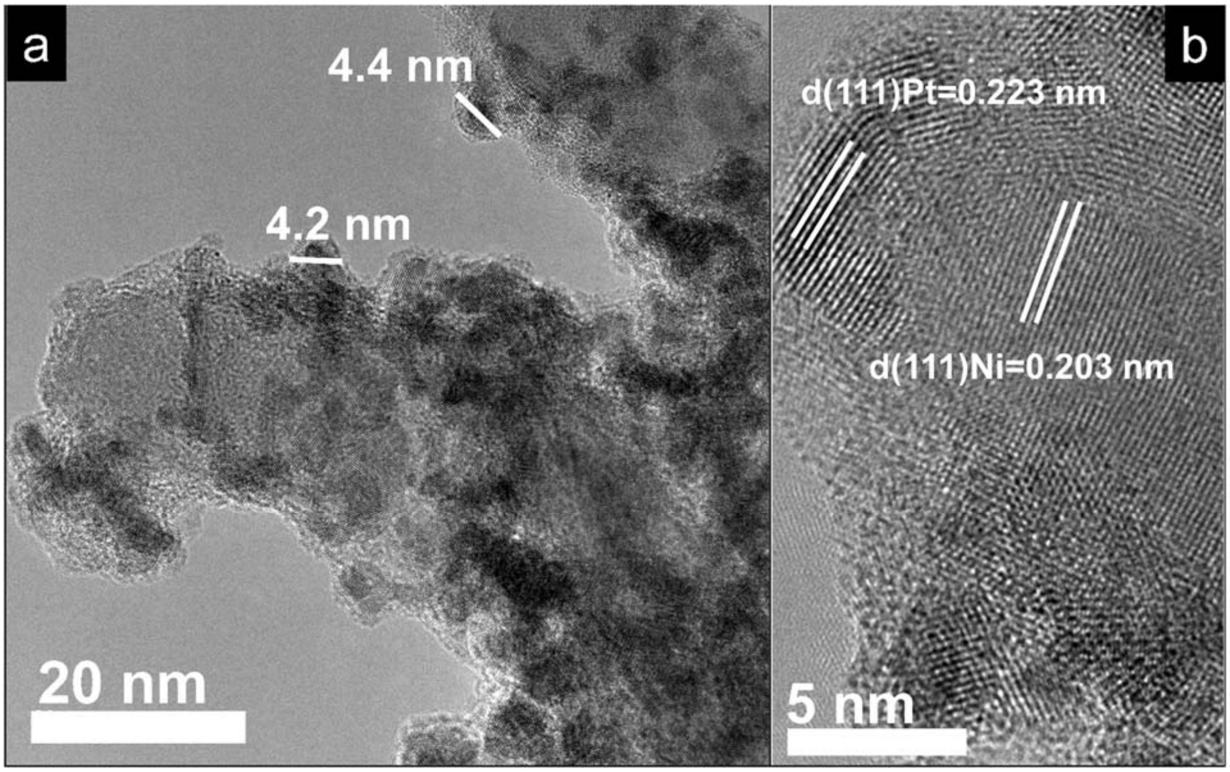

Fig. S10. TEM and HR-TEM of 5 Pt/Ni-SP, used for HER analysis for 50 ks at −1.5 V vs. Hg/HgO in 1-M NaOH.

Figure 11

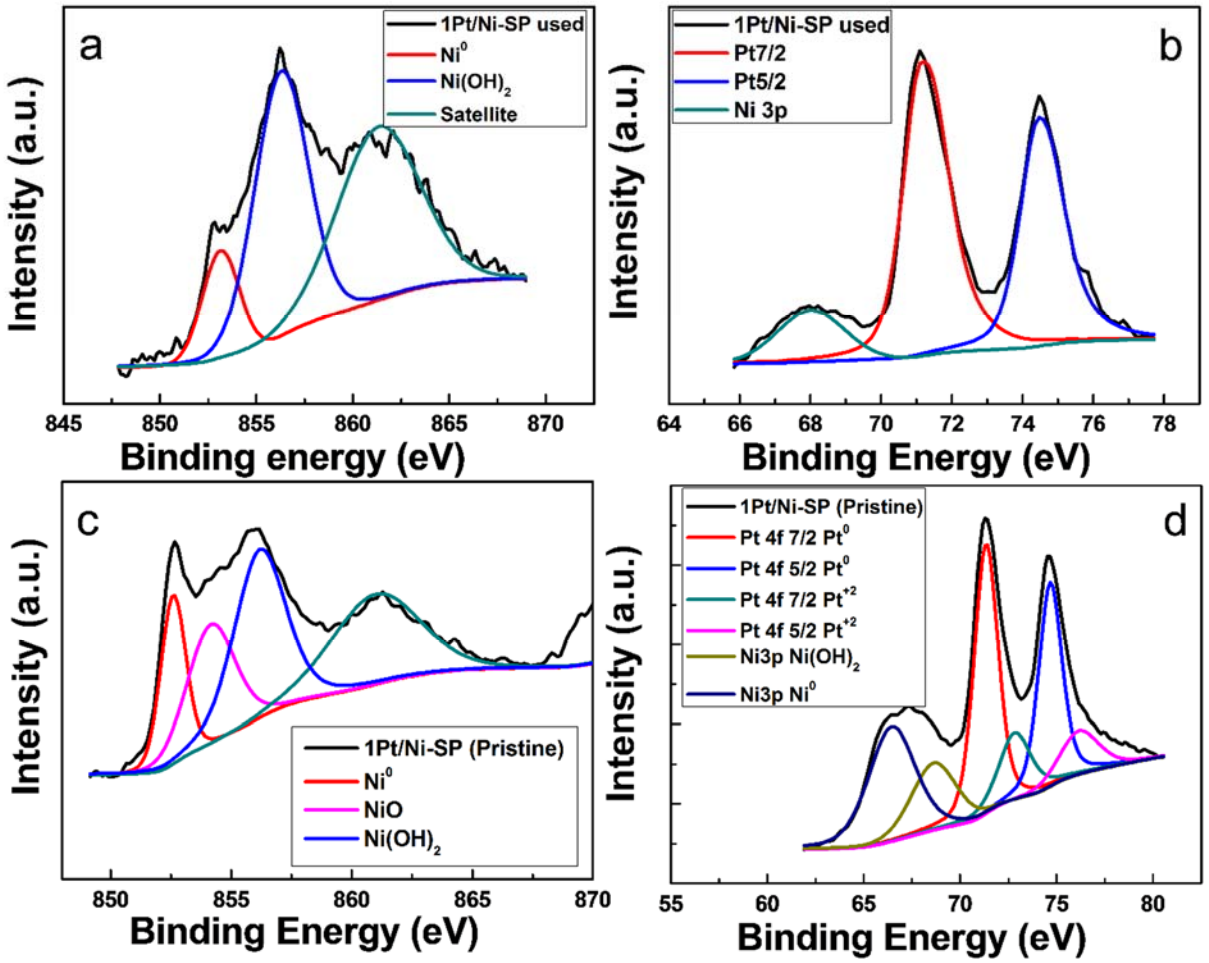

Fig. 11. XPS analysis of 1Pt/Ni-SP. (a) Ni phase and (b) Pt phase after electrochemical reaction for 50 ks at −1.5 V vs Hg/HgO, (c) and (d) show fresh prepared catalyst

Figure 12

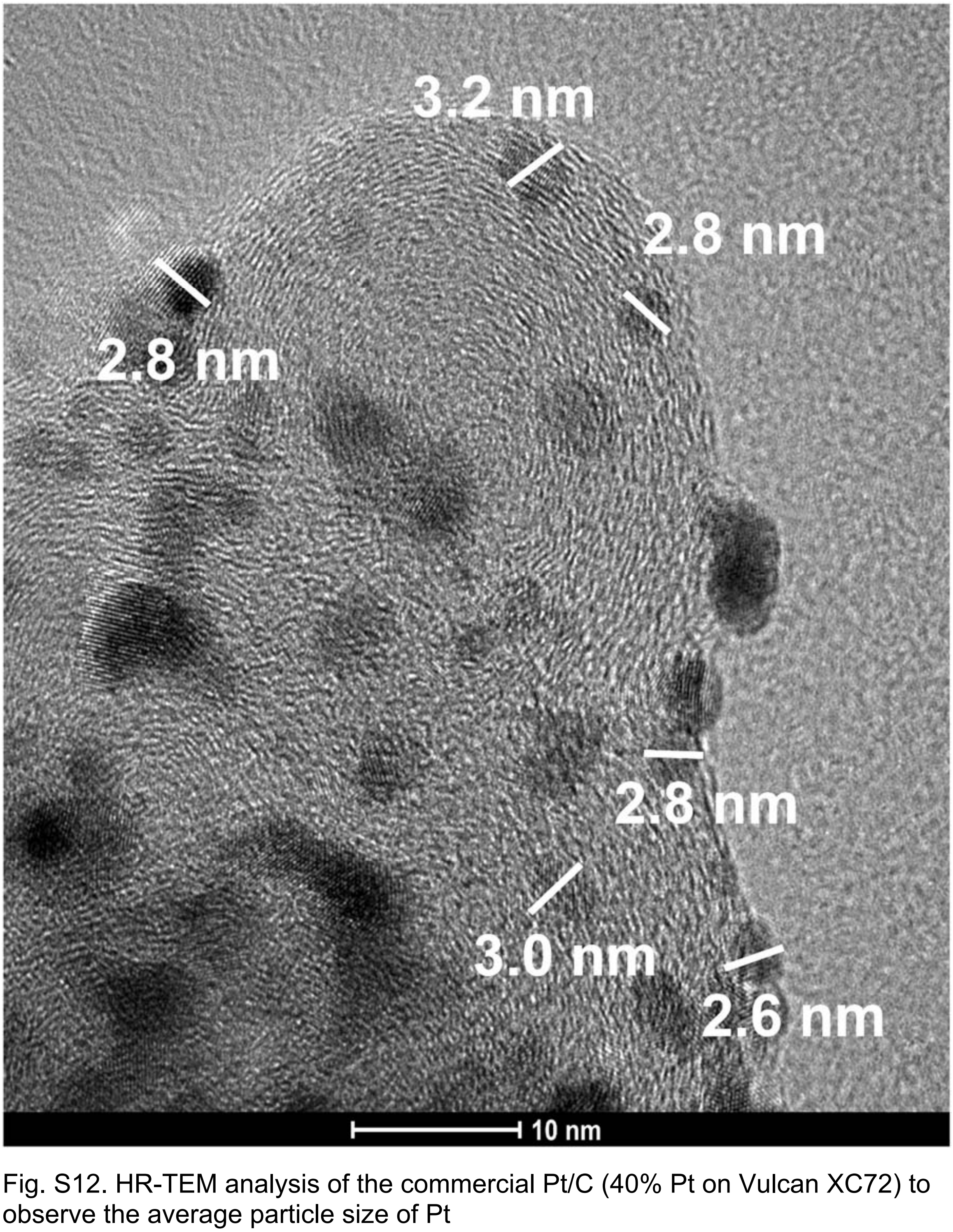

Fig. S12. HR-TEM analysis of the commercial Pt/C (40% Pt on Vulcan XC72) to observe the average particle size of Pt

Table S1. Results of ICP-OES analysis for the Pt/Ni-SP catalysts.

Sample	Theoretical Loading w/w%	ICP result w/w%
1Pt/Ni-SP	3.26	3.5
2Pt/Ni-SP	6.23	6.5
5Pt/Ni-SP	14.2	15.1

Electrochemical impedance analysis:

Electrochemical impedance spectroscopy (EIS) is performed in the range of 100 kHz to 0.1 Hz to observe the HER kinetics as shown in Fig. S13 and parameters are summarized in Table S2. EIS data was fitted in Armstrong-Henderson equivalent circuit where Rs is the resistance of solution, R_1 is the resistance of charge transfer and R_2 is the resistance of adsorbed hydrogen intermediates on the metal surface. Rs remained almost same in all the catalysts while the R_1 values are much smaller than R_2 values and remained similar in the case of Pt/Ni-SP and Pt/C catalysts that shows the Volmer reaction is not rate determining step. R_2 values decreases as the amount of Pt is increased in Pt/Ni-SP catalysts. R_2 valus of 2Pt/Ni-SP is very close to Pt/C value that corroborates with the Tafel and LSV results. In the case of Ni-SP, R_1 value is higher than R_2 value, that is an expected outcome as the rate-determining step in Ni is Volmer reaction. EIS is in complete agreement with LSV, Tafel slope and Chronoamperometric analysis.

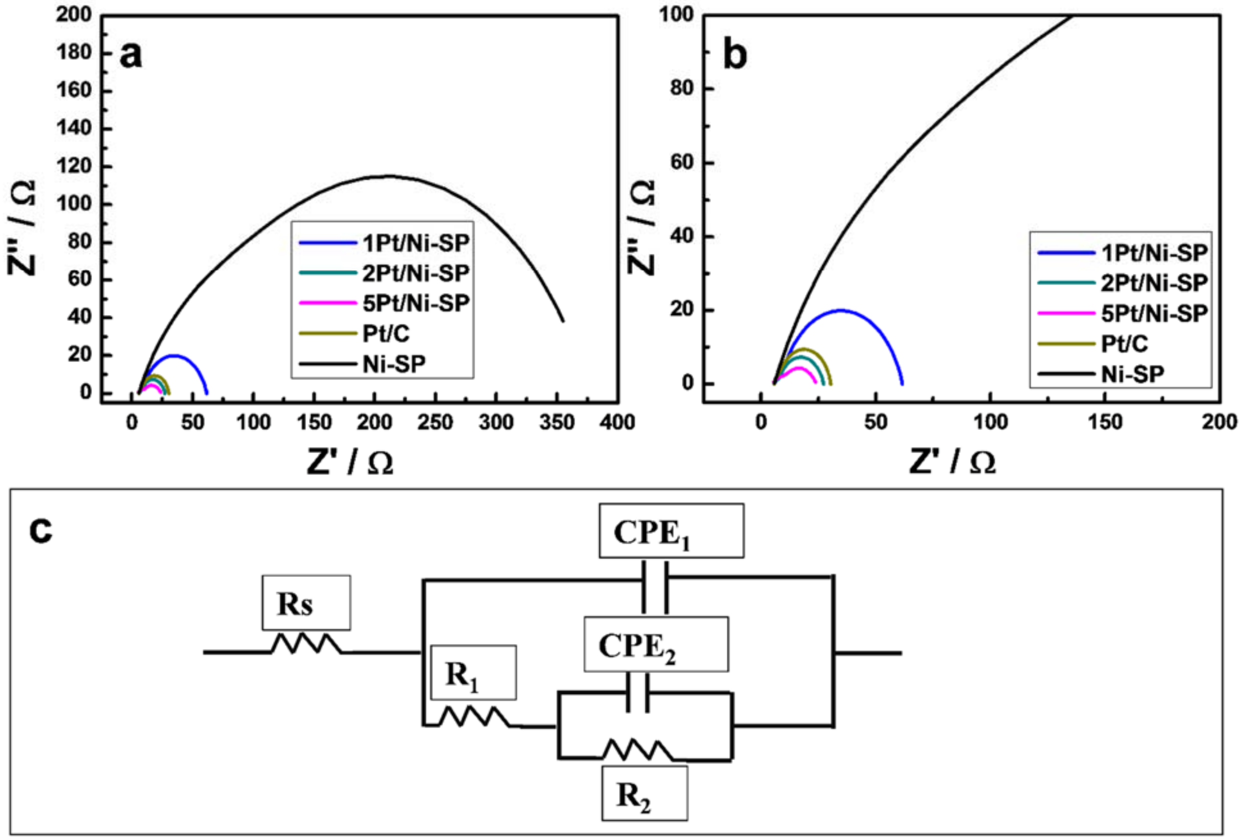

Fig. S13. (a) Nyquist plots at -1.0 V vs Hg/HgO, (b)) Nyquist plots at -1.0 V vs Hg/HgO in high frequency region, (c) Equivalent circuit diagram

Table S2. Parameters of impedance analysis performed in 1M NaOH at -1.0 V vs Hg/HgO

Catalyst	Rs (Ω)	R_1 (Ω)	R_2 (Ω)	CPE_1 (F)
1Pt/Ni-SP	5.5	5.6	50	0.00036
2Pt/Ni-SP	5.6	2.7	18.9	0.00013
5Pt/Ni-SP	5.2	6.6	13	0.00098
Pt/C	5.7	5.6	19	0.00038
Ni-SP	5.8	245.0	138.1	0.00052
